# Estimation of genetic diversity and population genetic structure in *Gymnema sylvestre* (Retz.) R. Br. ex Schult. populations using DAMD and ISSR markers

**DOI:** 10.1186/s43141-023-00497-7

**Published:** 2023-04-06

**Authors:** Kanchana Vaishnav, Vandana Tiwari, Anjala Durgapal, Baleshwar Meena, T. S. Rana

**Affiliations:** 1 Molecular Systematics Laboratory, CSIR-National Botanical Research Institute, Rana Pratap Marg, Lucknow, 226001 Uttar Pradesh India; 2 Maharana Pratap Government Degree College, Nanakmatta, Udham Singh Nagar, Kumaun University, Nainital, 263001 Uttarakhand India; 3 CSIR-Traditional Knowledge Digital Library Unit, New Delhi, 110067 India; 4 Academy of Scientific and Innovative Research (AcSIR), Ghaziabad, 201002 Uttar Pradesh India

**Keywords:** DAMD, *Gymnema sylvestre*, Genetic diversity, ISSR, Population genetic structure

## Abstract

**Background:**

*Gymnema sylvestre* (Retz.) R. Br. ex Schult. is a well-known medicinal plant against diabetes in India. There is as such no organized cultivation in India, and the plant is still being collected from the wild for their therapeutic uses. It is, therefore, important to estimate the genetic diversity and population genetic structure of *G. sylvestre* to ascertain the genetically diverse germplasm. The present study, therefore, was undertaken to analyze the genetic variability in 118 accessions belonging to 11 wild populations of *G. sylvestre* using directed amplification of minisatellite-region DNA (DAMD) and inter simple sequence repeats (ISSR).

**Results:**

The present genetic analyses of 11 populations with 25 markers (8 DAMD and 17 ISSR) revealed significant genetic diversity (*H* = 0.26, *I* = 0.40, *PPL* = 80.89%) at a species level, while the average genetic diversity at the population level was low. Among the 11 populations studied, PCH and UTK populations showed maximum genetic diversity, followed by KNR and AMB, while TEL population revealed the lowest genetic diversity. AMOVA and *G*_*st*_ values (0.18) revealed that most of the genetic variations are found within populations and very less among populations, and higher gene flow (*N*_*m*_ = 2.29) was found to be responsible for the genetic homogenization of the populations. The clustering pattern resulting from the UPGMA dendrogram was in congruence with STRUCTURE and PCoA, segregating all the 11 populations into two main genetic clusters: cluster I (populations of North and Central India) and cluster II (populations of South India). The clustering patterns obtained from all three statistical methods indicate that the genetic structure in *G. sylvestre* populations corresponds to the geographical diversity of the populations and represents a strong genetic structure.

**Conclusion:**

The genetically diverse populations identified during the present study could be a potential genetic resource for further prospecting and conserving this important plant resource.

**Supplementary Information:**

The online version contains supplementary material available at 10.1186/s43141-023-00497-7.

## Background

The genus *Gymnema* R. Br. (Apocynaceae) comprises approximately of 37 species worldwide [1], and about 14 species are found in India [2]. Among these, *Gymnema sylvestre* (Retz.) R. Br. ex Schult., commonly known as “Gurmar” or “Madhunashini,” is an important medicinal plant because of its reported anti-diabetic properties [3, 4]. It has a wide distribution range from tropical parts of Africa, Saudi Arabia, India, Sri Lanka, Southern China, and Southeast Asia to tropical Australia [5]. In India, it is reported to be distributed in the entire Peninsular region and has extended to Uttar Pradesh and Bihar [5, 6], covering tropical moist and dry deciduous, tropical thorn, tropical semi-evergreen, and tropical wet and dry evergreen forests [7]. *G. sylvestre* is a perennial, slow-growing, woody climber, with very small pale yellow-colored flowers in lateral umbel cyme that reaches up to 8–10 m height or more on the host plant. The presence of various triterpenoids such as gymnemic acid, gymnemasaponins, gymnemosides, gymnemanol, gymnemagenin, and gymnemasin along with several other glycosides such as gymsyloside F and gymsyloside G makes it a valuable plant in pharmaceuticals [8, 9]. Apart from these, its other major phytoconstituents are alkaloid drugs, anthracene derivatives, coumarin drugs, essential oils, flavonoids, valepotriates, etc. [10]. Besides anti-diabetic properties, *G. sylvestre* also has hypolipidemic, hepatoprotective [11], neuroprotective [12], immunomodulatory [13], cardioprotective [14], anti-inflammatory, antioxidant [15], anti-cancerous [16], antibacterial [17], and antiviral properties [18]. *G. sylvestre* has been used as an important ingredient in the preparations of many poly-herbal formulations for diabetes such as D-400 or Diabecon [19], IME-9 [20], and BGR-34 [21].

*G. sylvestre* is one of the 242 highly traded medicinal plants in India. The increased commercial demand for this highly valuable plant species has resulted in its overexploitation by local traders, and a big threat is operating on the species in the wild [22]. In addition to this main concern, the species is under pressure from habitat loss, invasion by exotic species, deterioration of the environment, and climate change. Also, the natural regeneration process of the species is slow and difficult due to low viability and poor germination rate of seed [23]. According to the Conservation Assessment and Management Prioritization (CAMP) exercises, *G. sylvestre* has been declared near threatened (NT) in Maharashtra, vulnerable (VU) in West Bengal, Madhya Pradesh, Chhattisgarh, Andhra Pradesh, and endangered (EN) in Rajasthan [22]. It is therefore critical to investigate comprehensively the patterns of genetic diversity and population structure in this medicinally important plant species so that appropriate conservation strategies and management programs can be undertaken.

There are many molecular markers available these days to analyze the genetic diversity and population genetic structure in plants. The most frequently used molecular markers are directed amplification of minisatellite-region DNA (DAMD) [24] and inter simple sequence repeats (ISSR) [25]. The main advantages of using these methods are that they are quick, inexpensive, robust, reproducible, highly polymorphic, randomly distributed throughout the genomes, and widely applicable to any genome [24–26]. These methods have been effectively used in the genetic diversity studies of many medicinal plant species like *Ephedra foliata* [27] *Curcuma* [28], *Bergenia ciliata* [29], and *Illicium griffithii* [26]. ISSR markers have also been found useful in unraveling the genetic diversity in some members like *Leptadenia pyrotechnica* [30], *Rauwolfia tetraphylla* [31], and *Decalepis salicifolia* [32] of the family Apocynaceae.

There are a few reports available on the genetic diversity of *G. sylvestre* using molecular markers [33–40]. There is, however, no comprehensive information available on the genetic diversity and population genetic structure of the species based on DAMD and ISSR methods. To the best of our knowledge, the present study on genetic diversity and population genetic structure of *G. sylvestre* using a combination of DAMD and ISSR markers seems to be a maiden attempt.

## Methods

### Plant materials and DNA extraction

**Fig. 1:**
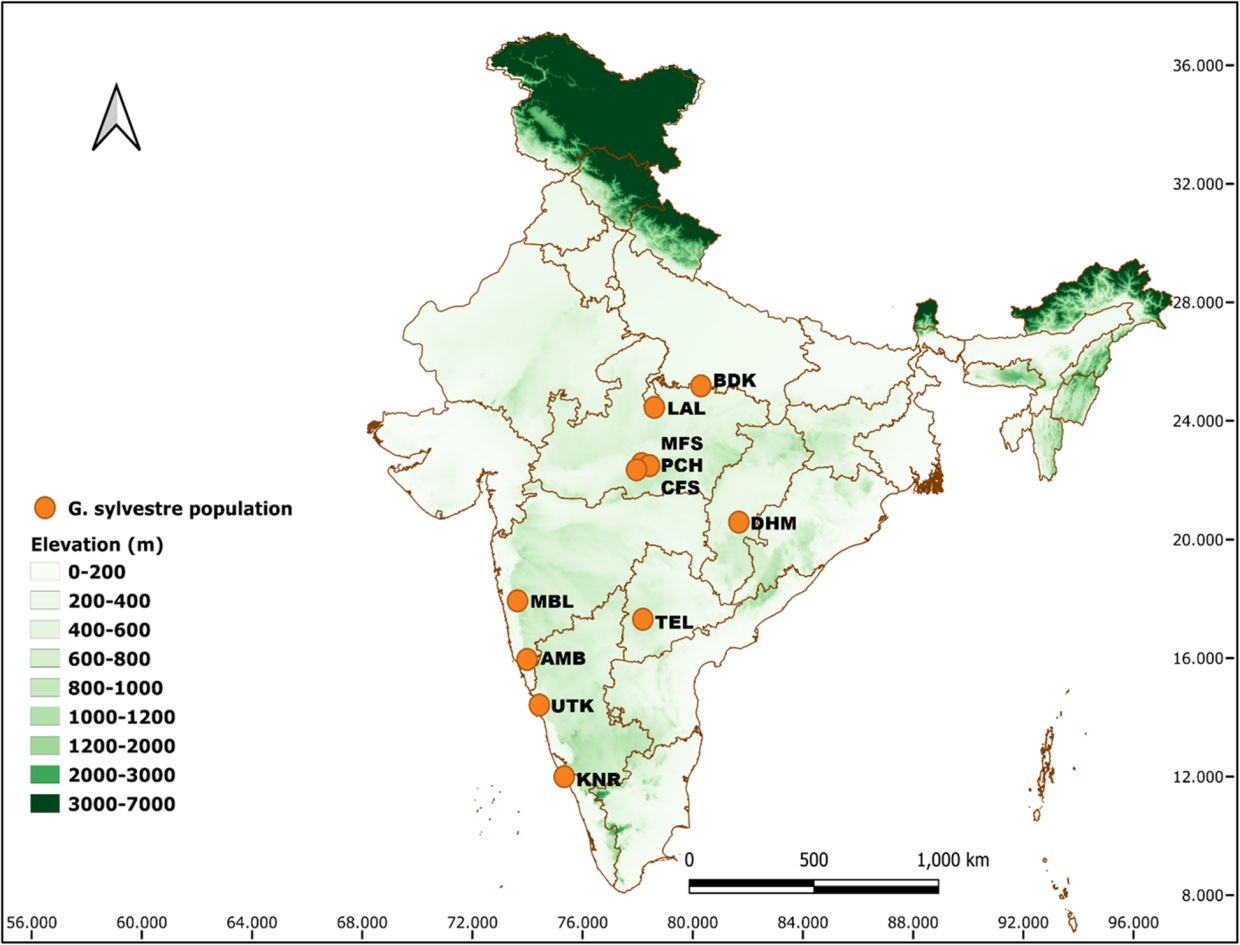
Location map prepared using QGIS v3.14 “Pi” for the collected wild populations of *G. sylvestre* in India. Population codes are as given in the Table 1

Plant samples were collected from seven Indian states (Uttar Pradesh, Madhya Pradesh, Chhattisgarh, Maharashtra, Telangana, Karnataka, and Kerala). In the present analysis, we have considered a total of 118 accessions of 11 populations of *G. sylvestre*. A closely related species *Tylophora indica* (Burm. f.) Merr*.* was used as the out-group (Table 1, Fig. 1). The geocoordinates such as latitude (°N), longitude (°E), and elevation (m) for each population were recorded with the help of a global positioning system receiver (Garmin, USA). Fresh leaf samples were cleaned and stored dry at room temperature using self-indicating blue silica gel. Voucher specimens of all samples were prepared and have been deposited in the herbarium of CSIR-National Botanical Research Institute (LWG), Lucknow, Uttar Pradesh, India.

**Table 1:** Details of wild populations of *G. sylvestre* collected from different locations of India

SN	Population code	Localities	Latitude (°N)	Longitude (°E)	Elevation (m)	Sample size	Collection number
1	BDK	Mahoba and Chitrakoot, Bundelkhand, Uttar Pradesh	25° 10′ 45.08″	80° 17′ 46.65″	222	7	1–7
2	LAL	Lalitpur, Bundelkhand, Uttar Pradesh	24° 27′ 17.51″	78° 36′ 22.89″	383	11	8–18
3	MFS	Madai forest, Satpura Tiger Reserve, Madhya Pradesh	22° 33′ 24.42″	78° 8′ 40.56″	371	9	19–27
4	PCH	Pachmarhi, Madhya Pradesh	22° 29′ 14.18″	78° 25′ 47.86″	905	16	28–43
5	CFS	Churna forest, Satpura Tiger Reserve, Madhya Pradesh	22° 20′ 53.67″	77° 57′ 36.44″	385	7	44–50
6	DHM	Keregaon, Dhamtari, Chhattisgarh	20° 35′ 28.85″	81° 40′ 22.63″	348	10	51–60
7	MBL	Mahabaleshwar, Maharashtra	17° 56′ 6.06″	73° 38′ 12.37″	1094	11	61–71
8	TEL	Hyderabad and Vikarabad, Telangana	17° 18′ 47.92″	78° 11′ 22.92″	24	7	72–78
9	AMB	Amboli, Maharashtra	15° 57′ 19.62″	73° 58′ 59.47″	608	14	79–92
10	UTK	Uttar Kannada, Karnataka	14° 25′ 1.41″	74° 25′ 54.87″	59	19	93–111
11	KNR	Kannur, Kerala	11° 59′ 59.25″	75° 19′ 56.63″	52	7	112–118
	Total					118

Total genomic DNA from silica-dried leaf tissues was isolated using the CTAB method [41]. The quality of the extracted DNA was checked on 0.8% agarose gel, stained with ethidium bromide and compared with a set of known concentrations of lambda DNA (double digested with EcoRI and HindIII) using UV light photography in UVITEC gel documentation system (MilliporeSigma, USA), and quantity was measured by UV spectroscopy using a Nanodrop UV Spectrophotometer (Thermo Scientific, USA).

### PCR amplification using DAMD and ISSR markers

The 20 DAMD markers available in the public domain [24] were custom synthesized from Sigma Aldrich Chemicals Private Limited, Bangalore, Karnataka, India. A set of 100 anchored ISSR markers was procured from the University of British Columbia, Canada. PCR amplification with DAMD and ISSR markers was carried out according to Zhou et al. [42].

All 20 DAMD markers were screened with three template DNAs of *G. sylvestre*, of which eight markers resulted in distinct and reproducible fragments that were subsequently used for further amplification of the entire set of 11 populations. The amplification with DAMD marker was carried out in a 20 μl reaction mixture containing 4 μl of template DNA (80 ng), 2 μl of 10 mM dNTP mix (2.5 mM each dNTP), 1.2 μl of 25 mM MgCl_2_, 2.5 μl of suitable 10 × assay buffer (*Taq* Buffer A) supplied along with the enzyme, 1.5 μl of 10 μM primer, and 0.33 μl of 3.0 U *Taq* DNA polymerase (Genei Laboratories Private Limited, Bangalore, Karnataka, India) using a Proflex PCR System (Applied Biosystems, Life Technologies, USA). After initial denaturation at 94 °C for 4 min, each cycle consisted of 1 min of denaturation at 94 °C, 2 min of annealing at (50 °C–55 °C), and 2.5 min extension at 72 °C, along with 7 min final extension at the end of 40 cycles. The amplification pattern of 118 *G. sylvestre* individuals is shown by the representative gel profile of marker FvIIex8C (Fig. S1a).

Similarly, 100 ISSR markers were screened with three template DNAs of *G. sylvestre*, of which 17 markers that resulted in distinct and reproducible fragments were finally selected for further amplification of the entire set of 11 populations. ISSR-PCR amplification of 2 μl of template DNA (40 ng) was carried out in a 20 μl reaction volume, containing 1 μl of 10 mM dNTP mix (2.5 mM each dNTP), 1 μl of 25 mM MgCl_2_, 2.5 μl of suitable 10 × assay buffer (*Taq* Buffer A) supplied along with the enzyme, 1.5 μl of 10 μM primer, and 0.33 μl of 3.0 U *Taq* DNA polymerase (Genei Laboratories Private Limited, Bangalore, Karnataka, India) using a Proflex PCR System (Applied Biosystems, Life Technologies, USA). After initial denaturation at 94 °C for 4 min, each cycle consisted of 1 min of denaturation at 94 °C, 1 min of annealing at (50 °C–54 °C), and 2 min extension at 72 °C along with 7 min final extension at the end of 35 cycles. The amplification pattern of 118 *G. sylvestre* individuals is shown by the representative gel profile of marker UBC-809 (Fig. S1b).

A total of 25 markers (8 DAMD and 17 ISSR) were finally selected and profiles of the complete set of 118 individuals of *G. sylvestre* and one individual of outgroup species *T. indica* were carried out with these 25 markers (Table 2). The amplified PCR products were loaded on 1.5% agarose gel, and stained with ethidium bromide, using 1X TBE buffer at a constant voltage of 5 V/cm. The size of each fragment for all the markers was determined using low range (100–3000 bp) DNA ruler plus (Genei Laboratories Private Limited, Bangalore, Karnataka, India). After electrophoresis, the gel was visualized using UVITech gel documentation system (Millipore Sigma, USA), and the patterns were photographed and documented as digital images.

**Table 2:** Details of DAMD and ISSR markers and the polymorphism obtained with these markers

Marker	Sequence (5′ → 3′)	AT (°C)	TL	ML	PL	PPL	Approx. loci size (bp)	PIC	Rp	DI	EMR	MI
DAMD												
14C2	GGCAGGATTGAAGC	50	17	3	14	82.35	350–1200	0.20	21.58			
33.6	AGGGCTGGAGG	50	21	2	19	90.48	170–1100	0.29	27.05			
FvIIex8	ATGCACACACACAGG	55	21	6	15	71.43	230–1000	0.23	30.51			
FvIIex8C	CCTGTGTGTGTGCAT	55	19	3	16	84.21	275–1300	0.30	25.19			
HBV	GGTGTAGAGAGGGGT	50	17	2	15	88.24	360–1200	0.24	21.44			
HVR	CCTCCTCCCTCCT	50	19	4	15	78.95	310–1300	0.22	22.81			
URP9F	ATGTGTGCGATCAGTTGCTG	55	18	3	15	83.33	200–1100	0.23	24.61			
URP38F	AAGAGGCATTCTACCACCAC	55	23	4	19	82.61	200–1600	0.22	37.86			
Total (8)			155	27	128		170–1600					
Average/marker			19.38	3.38	16	82.58		0.24	26.38	0.29	13.21	3.88
ISSR												
UBC-808	AGAGAGAGAGAGAGAGC	52	13	2	11	84.62	240–1400	0.12	23.17			
UBC-809	AGAGAGAGAGAGAGAGG	54	17	5	12	70.59	290–1000	0.22	21.34			
UBC-810	GAGAGAGAGAGAGAGAT	50	13	2	11	84.62	300–1000	0.29	15.83			
UBC-812	GAGAGAGAGAGAGAGAA	50	16	5	11	68.75	240–1000	0.20	23.95			
UBC-823	TCTCTCTCTCTCTCTCC	50	19	4	15	78.95	280–1500	0.20	28.83			
UBC-825	ACACACACACACACACT	50	15	2	13	86.67	300–1100	0.22	20.25			
UBC-826	ACACACACACACACACC	58	20	3	17	85.00	350–1250	0.26	30.35			
UBC-830	TGTGTGTGTGTGTGTGG	58	18	3	15	83.33	300–1200	0.32	24.13			
UBC-835	AGAGAGAGAGAGAGAGYC	50	22	3	19	86.36	300–1500	0.27	30.83			
UBC-836	AGAGAGAGAGAGAGAGYA	54	15	2	13	86.67	290–820	0.24	20.78			
UBC-840	GAGAGAGAGAGAGAGAYT	50	11	1	10	90.91	300–900	0.21	13.05			
UBC-841	GAGAGAGAGAGAGAGAYC	51	16	3	13	81.25	270–1200	0.26	19.51			
UBC-842	GAGAGAGAGAGAGAGAYG	54	18	4	14	77.78	190–950	0.33	23.91			
UBC-860	TGTGTGTGTGTGTGTGRA	54	19	1	18	94.74	350–1200	0.28	21.46			
UBC-866	CTCCTCCTCCTCCTCCTC	55	12	6	06	50.00	500–1200	0.22	19.12			
UBC-868	GAAGAAGAAGAAGAAGAA	53	14	4	10	71.43	310–1000	0.18	21.66			
UBC-887	DVDTCTCTCTCTCTCTC	50	16	5	11	68.75	300–1000	0.23	23.29			
Total (17)			274	55	219		190–1500					
Average/marker			16.12	3.24	12.88	79.93		0.24	22.44	0.30	10.30	3.14
Cumulative (25)			429	82	347	80.89	170–1600	0.24	24.41	0.30	11.76	3.51

*TL* total number of loci amplified, *ML* monomorphic loci, *PL* polymorphic loci, *PPL* percentage of polymorphic loci, *Approx.* approximately, *bp* base pairs, *PIC* polymorphic information content, *Rp* resolving power, *DI* diversity index, *EMR* effective multiplex ratio, *MI* marker index. Mixed bases; D = A or G or T not C; R = A or G; V = A or C or G not T; Y = C or T

### Scoring of PCR profiles and data analyses

Data analysis with the two markers (8 DAMD and 17 ISSR) was carried out for all 25 reproducible profiles showing consistent banding patterns. A binary data matrix was generated indicating the presence (1) or absence (0) of a band for each marker in the assay. The data matrices (DAMD, ISSR, and cumulative; DAMD + ISSR) were analyzed statistically to evaluate the relative informativeness of the two markers independently and cumulatively, by calculating the percentage of polymorphic loci (PPL), polymorphic information content (PIC), resolving power (Rp), diversity index (DI), effective multiplex ratio (EMR), and marker index (MI) for each marker [25, 43, 44]. Mantel *Z*-statistics to test the correlation (*r*) between the three pairs of datasets (cumulative v/s DAMD, cumulative v/s ISSR, and DAMD v/s ISSR) and to ascertain goodness of fit between these markers computed using MXCOMP module of NTSYS-pc software version 2.02e [45]. Sequential agglomerative hierarchical nested (SAHN) clustering module [46] using the unweighted pair group method with arithmetic mean (UPGMA) method for both individual marker and cumulative marker data was carried out in NTSYS-pc software version 2.02e to generate the UPGMA tree of *G. sylvestre* individuals with the out-group species which was visualized in MEGA11 software [47]. Genetic similarity and distances based on Jaccard’s coefficient [48] were calculated using neighbor-joining (NJ) and UPGMA in the FREETREE program version 0.9.1.5 [49] both by individual marker system and cumulatively.

The 11 populations considered in the present study were assumed to be in Hardy–Weinberg equilibrium for estimating the parameters of genetic diversity at population and species levels. The various genetic diversity parameters viz., *PPL*, observed number of alleles (*N*_*a*_), effective number of alleles (*N*_*e*_), Nei’s gene diversity (*H*) [50], Shannon information index (*I*) [51], total genetic diversity (*H*_*t*_), genetic diversity within populations (*H*_*s*_), Nei’s genetic differentiation coefficient among populations (*G*_*st*_), and gene flow among populations (*N*_*m*_) were calculated in POPGENE program version 1.32 [52] using cumulative marker data. Besides this, the population dendrogram based on Nei’s original measures of genetic distances [53] using the UPGMA method was also calculated in POPGENE program using cumulative marker data. The UPGMA dendrogram was visualized in FigTree version 1.4.4 software [54].

Hierarchical partitioning of genetic variability at three strata, i.e., within populations, among populations, and among regions were examined by analysis of molecular variance (AMOVA) and clustering of populations in principal coordinate analysis (PCoA) for spatial representation of the relative genetic distances among all the individuals of *G. sylvestre* were done in GenAlEx program version 6.5 [55] using cumulative marker data. A Mantel test [56] was also conducted in GenAlEx program at 9999 permutations to determine the correlation (*r*) between pair-wise geographic and genetic distances of 11 populations and to evaluate the isolation by distance (IBD) model using cumulative marker data.

A cumulative dataset consisting of a set of individuals genotyped using multilocus markers was evaluated for clustering of populations using the Bayesian approach (Markov chain Monte Carlo (MCMC)) implanted in STRUCTURE software version 2.3 [57, 58]. This quantitative clustering method stratifies the populations by computing the proportion of the genome of an individual originating from each inferred population. For analysis, the number of *K* with prior population information was inferred using the admixture model with correlated allele frequencies. The program was run 20 times for each “*K*,” ranging from 1 to 12. Each run consisted of a burn-in time of 50,000 and a MCMC replication of 150,000. The number of *K* was obtained in a web-based python program STRUCTURE HARVESTER version 0.6.94 [59] by implementing the method described by Evanno et al. [60]. STRUCTURE HARVESTER generated plots comprise the mean of estimated Ln probability of data, Evanno results showing the mean rate of change of the likelihood distribution, mean of the absolute value of second order rate of change of the likelihood distribution, and the number of “*K*” groups that best fit the data.

## Results

### DAMD and ISSR polymorphism

A total of 155 loci were amplified with 8 DAMD markers in 118 individuals of 11 populations, with an average of 19.38 loci per marker. Out of these 155 loci, 128 were polymorphic corresponding to 82.58% polymorphism, with an average of 16 polymorphic loci per marker. The size of amplified products varied approximately from 170 to 1600 bp. Among the 8 DAMD markers, 33.6 showed maximum polymorphism (90.48%), whereas the FvIIex8 showed minimum (71.43%). The marker URP38F came up with the maximum Rp value (37.86), whereas HBV with minimum (21.44). The PIC value was found to be maximum (0.30) for the marker FvIIex8c, whereas minimum (0.20) for 14C2. Among the 8 DAMD markers, two DAMD markers were found to be reasonably informative with PIC values > 0.25 and < 0.50 (33.6 = 0.29, FvIIex8c = 0.30), whereas the remaining six markers were slightly informative with their PIC values < 0.25 (14C2 = 0.20, FvIIex8 = 0.23, HBV = 0.24, HVR = 0.22, URP9F = 0.23 and URP38F = 0.22; Table 2).

A total of 274 loci were obtained with 17 ISSR markers with an average of 16.12 loci per marker. Out of these 274 loci, 219 were polymorphic corresponding to 79.93% polymorphism, with an average of 12.88 polymorphic loci per marker. The size of amplified products varied from 190 to 1500 bp. Among the 17 ISSR markers, UBC-860 showed maximum polymorphism (94.74%) whereas the UBC-866 showed minimum (50.00%). The marker UBC-835 came up with the maximum Rp value (30.83), whereas UBC-840 with minimum (13.05). The PIC value was found to be maximum (0.33) for the marker UBC-842 whereas minimum (0.12) for UBC-808. Out of 17 ISSR markers, seven markers were found to be reasonably informative with PIC values > 0.25 and < 0.50 (UBC-810 = 0.29, UBC-826 = 0.26, UBC-830 = 0.32, UBC-835 = 0.27, UBC-841 = 0.26, UBC-842 = 0.33, UBC-860 = 0.28), whereas rest of the ten markers were slightly informative with their PIC values < 0.25 (UBC-808 = 0.12, UBC-809 = 0.22, UBC-812 = 0.20, UBC-823 = 0.20, UBC-825 = 0.22, UBC-836 = 0.24, UBC-840 = 0.21, UBC-866 = 0.22, UBC-868 = 0.18, UBC-887 = 0.23; Table 2).

The PIC and DI values calculated for each marker system (DAMD: PIC = 0.24, DI = 0.29; ISSR: PIC = 0.24, DI = 0.30) were found almost equal for both the marker systems. However, the Rp, EMR, and MI values of the DAMD were found to be higher (Rp = 26.38, EMR = 13.21, MI = 3.88) than the ISSR (Rp = 22.44, EMR = 10.30, MI = 3.14) indicating the higher informativeness of DAMD over ISSR markers in estimating the genetic diversity of *G. sylvestre*. Cumulative analysis with 25 markers (8 DAMD + 17 ISSR) revealed 80.89% polymorphism with PIC, Rp, DI, EMR, and MI values of 0.24, 24.41, 0.30, 11.76, and 3.51, respectively (Table 2). The Mantel test performed for three pairs of datasets (DAMD v/s cumulative, ISSR v/s cumulative, and DAMD v/s ISSR) revealed a perfect positive correlation (*r* = 1) for each pair of data sets. The Mantel test indicates the effectiveness and suitability of both marker systems in estimating the genetic diversity and population genetic structure in natural populations of *G. sylvestre* (Table S1).

### Analysis of genetic diversity

After considering and analyzing the various genetic diversity parameters viz., *N*_*a*_, *N*_*e*_, *H*, *I*, and *PPL*; the PCH (*N*_*a*_ = 1.59, *N*_*e*_ = 1.35, *H* = 0.20, *I* = 0.30, *PPL* = 58.74%) and UTK population (*N*_*a*_ = 1.59, *N*_*e*_ = 1.34, *H* = 0.20, *I* = 0.30, *PPL* = 58.51%) were found to be the most diverse, closely followed by KNR (*N*_*a*_ = 1.51, *N*_*e*_ = 1.36, *H* = 0.20, *I* = 0.29, *PPL* = 51.28%) and AMB population (*N*_*a*_ = 1.52, *N*_*e*_ = 1.33, *H* = 0.19, *I* = 0.28, *PPL* = 51.52%), whereas TEL population was found to be least diverse (*N*_*a*_ = 1.29, *N*_*e*_ = 1.19, *H* = 0.11, *I* = 0.16, *PPL* = 29.37%) among 11 populations of *G. sylvestre* (Table 3).

**Table 3:** Intra-population diversity statistics of *G. sylvestre* populations computed in POPGENE software

Population (sample size)	Mean *N*_*a*_ (SD)	Mean *N*_*e*_ (SD)	Mean *H* (SD)	Mean* I* (SD)	NPL	*PPL*	Mean *H*_*t*_ (SD)	Mean *H*_*s*_ (SD)	*G*_*st*_	*N*_*m*_
BDK (7)	1.35 (0.48)	1.24 (0.37)	0.13 (0.20)	0.20 (0.28)	151	35.20				
LAL (11)	1.39 (0.49)	1.24 (0.36)	0.14 (0.20)	0.21 (0.28)	168	39.16				
MFS (9)	1.47 (0.50)	1.29 (0.37)	0.17 (0.20)	0.25 (0.29)	203	47.32				
PCH (16)	1.59 (0.49)	1.35 (0.37)	0.20 (0.20)	0.30 (0.29)	252	58.74				
CFS (7)	1.43 (0.50)	1.27 (0.38)	0.16 (0.20)	0.23 (0.29)	184	42.89				
DHM (10)	1.47 (0.50)	1.31 (0.39)	0.18 (0.21)	0.26 (0.29)	203	47.32				
MBL (11)	1.35 (0.48)	1.22 (0.35)	0.13 (0.19)	0.19 (0.28)	150	34.97				
TEL (7)	1.29 (0.46)	1.19 (0.34)	0.11 (0.18)	0.16 (0.26)	126	29.37				
AMB (14)	1.52 (0.50)	1.33 (0.38)	0.19 (0.20)	0.28 (0.29)	221	51.52				
UTK (19)	1.59 (0.49)	1.34 (0.37)	0.20 (0.20)	0.30 (0.29)	251	58.51				
KNR (7)	1.51 (0.50)	1.36 (0.41)	0.20 (0.22)	0.29 (0.30)	220	51.28				
Average	1.46	1.29	0.16	0.24	193.5	45.12				
Total (118)	1.81 (0.39)	1.44 (0.36)	0.26 (0.19)	0.40 (0.25)	347	80.89	0.26 (0.03)	0.21 (0.02)	0.18	2.29

*N*_*a*_ observed number of alleles, *N*_*e*_ effective number of alleles, *H* Nei’s gene diversity, *I* Shannon’s information index, *NPL* number of polymorphic loci, *PPL* percentage of polymorphic loci, *H*_*t*_ total genetic diversity, *H*_*s*_ genetic diversity within populations, *G*_*st*_ Nei’s genetic differentiation coefficient among populations, *Nm* gene flow among populations, *SD* standard deviation of mean values. Population codes are as given in Table 1

The total genetic diversity of the *G. sylvestre* was found significantly high (*N*_*a*_ = 1.81, *N*_*e*_ = 1.44, *H* = 0.26, *I* = 0.40, *PPL* = 80.89%), but the average genetic diversity of a population (*N*_*a*_ = 1.46, *N*_*e*_ = 1.29, *H* = 0.16, *I* = 0.24) was found much lower than the genetic diversity at the species level. This could be due to the differences in genetic diversity of different populations, from PCH and UTK (*PPL* = 59%) to TEL (*PPL* = 29.37%) with an average of 45.12% polymorphism per population. In the total genetic diversity of *G. sylvestre* (*H*_*t*_ = 0.26), most of the part was found to be contributed by genetic diversity within populations (*H*_*s*_ = 0.21; Table 3).

The region-wise genetic diversity analysis revealed that the South Indian region which comprised of total five populations showed the highest genetic diversity (*N*_*a*_ = 1.73, *N*_*e*_ = 1.39, *H* = 0.24, *I* = 0.36, and *PPL* = 72.73%) closely followed by Central-Indian region with four populations (*N*_*a*_ = 1.69, *N*_*e*_ = 1.36, *H* = 0.22, *I* = 0.33, and *PPL* = 69.00%), while the North-Indian region with two populations showed the lowest genetic diversity (*N*_*a*_ = 1.47, *N*_*e*_ = 1.26, *H* = 0.15, *I* = 0.23, and *PPL* = 46.85%; Table S2).

### Analysis of population genetic structure

The hierarchical partitioning of the total genetic variations of the species by AMOVA affirmed most of the genetic variations within populations (62%), followed by among regions (22%), and low among populations (16%) (Table 4). This result was further supported by *G*_*st*_ among 11 natural populations of *G. sylvestre* (0.18), which indicates that of the total genetic variations of species, there are only 18% genetic variations among populations, and most of the genetic variations, i.e., 82%, are within populations. This means that these 11 populations are genetically less differentiated from each other (Table 3). This was further substantiated by the significant value of gene flow (*N*_*m*_ = 2.29) among 11 populations which is responsible for lowering the genetic differentiation among populations (Table 3). The population genetic structure of *G. sylvestre* was also revealed by the Nei’s gene or genetic diversity value, as of the total genetic diversity of the species (*H*_*t*_ = 0.26), most of the part (*H*_*s*_ = 0.21; 80.77%) was found to be occupied by genetic diversity within populations (Table 3).

**Table 4:** AMOVA analysis carried out using GenAlEx program for the cumulative^a^ (DAMD and ISSR) data of 118 individuals of *G. sylvestre*

Source of variations	Degree of freedom	Sum of squares	Mean of squares	Variance component	Percentage of variations
Among regions	2	1223.823	611.912	13.162	22%
Among populations	8	1064.946	133.118	9.284	16%
Within populations	107	3861.883	36.092	36.092	62%
Total	117	6150.653		58.538	100%

^a^Combined DAMD and ISSR data matrices

The inter-population genetic identities ranged from 0.8072 (BDK v/s KNR) to 0.9627 (BDK v/s LAL) and the inter-population genetic distances ranged from 0.0380 (BDK and LAL) to 0.2142 (BDK v/s KNR). From these values, the genetic identities and genetic distances are found to be congruent with the geographical distances between populations (Table 5). Mantel test carried out for calculating the correlation between pairwise geographic and genetic distances in *G. sylvestre* populations revealed a very strong positive correlation (*r* = 0.831, *p ˂ 0.0001*) between geographic distance and genetic distance, indicating that the current genetic structure of the *G. sylvestre* populations is according to the geographical distances and isolation between populations (Fig. S2).

**Table 5:** Inter-population Nei’s original measures of genetic identity and genetic distance calculated among populations of *G. sylvestre* using the POPGENE software

Population code	BDK	LAL	MFS	PCH	CFS	DHM	MBL	TEL	AMB	UTK	KNR
BDK	****	**0.9627**	0.9194	0.9382	0.9225	0.9252	0.8200	0.8266	0.8242	0.8307	**0.8072**
LAL	**0.0380**	****	0.9280	0.9454	0.9275	0.9225	0.8260	0.8407	0.8411	0.8409	0.8163
MFS	0.0840	0.0748	****	0.9570	0.9484	0.9326	0.8301	0.8378	0.8619	0.8533	0.8176
PCH	0.0638	0.0561	0.0440	****	0.9406	0.9406	0.8415	0.8574	0.8643	0.8709	0.8423
CFS	0.0807	0.0753	0.0530	0.0612	****	0.9355	0.8159	0.8232	0.8510	0.8474	0.8141
DHM	0.0778	0.0807	0.0698	0.0612	0.0667	****	0.8249	0.8451	0.8609	0.8571	0.8344
MBL	0.1984	0.1911	0.1862	0.1726	0.2034	0.1925	****	0.8497	0.8866	0.8820	0.8522
TEL	0.1904	0.1735	0.1769	0.1539	0.1945	0.1683	0.1629	****	0.8959	0.8858	0.8575
AMB	0.1933	0.1730	0.1486	0.1459	0.1613	0.1498	0.1203	0.1099	****	0.9538	0.9050
UTK	0.1855	0.1733	0.1587	0.1382	0.1656	0.1542	0.1255	0.1213	0.0473	****	0.9348
KNR	**0.2142**	0.2030	0.2014	0.1716	0.2057	0.1810	0.1599	0.1537	0.0999	0.0674	****

Nei’s genetic identity (above diagonal) and genetic distance (below diagonal). The maximum and minimum genetic identities and genetic distances are highlighted in bold. Population codes are as given in Table 1

**Fig. 2:**
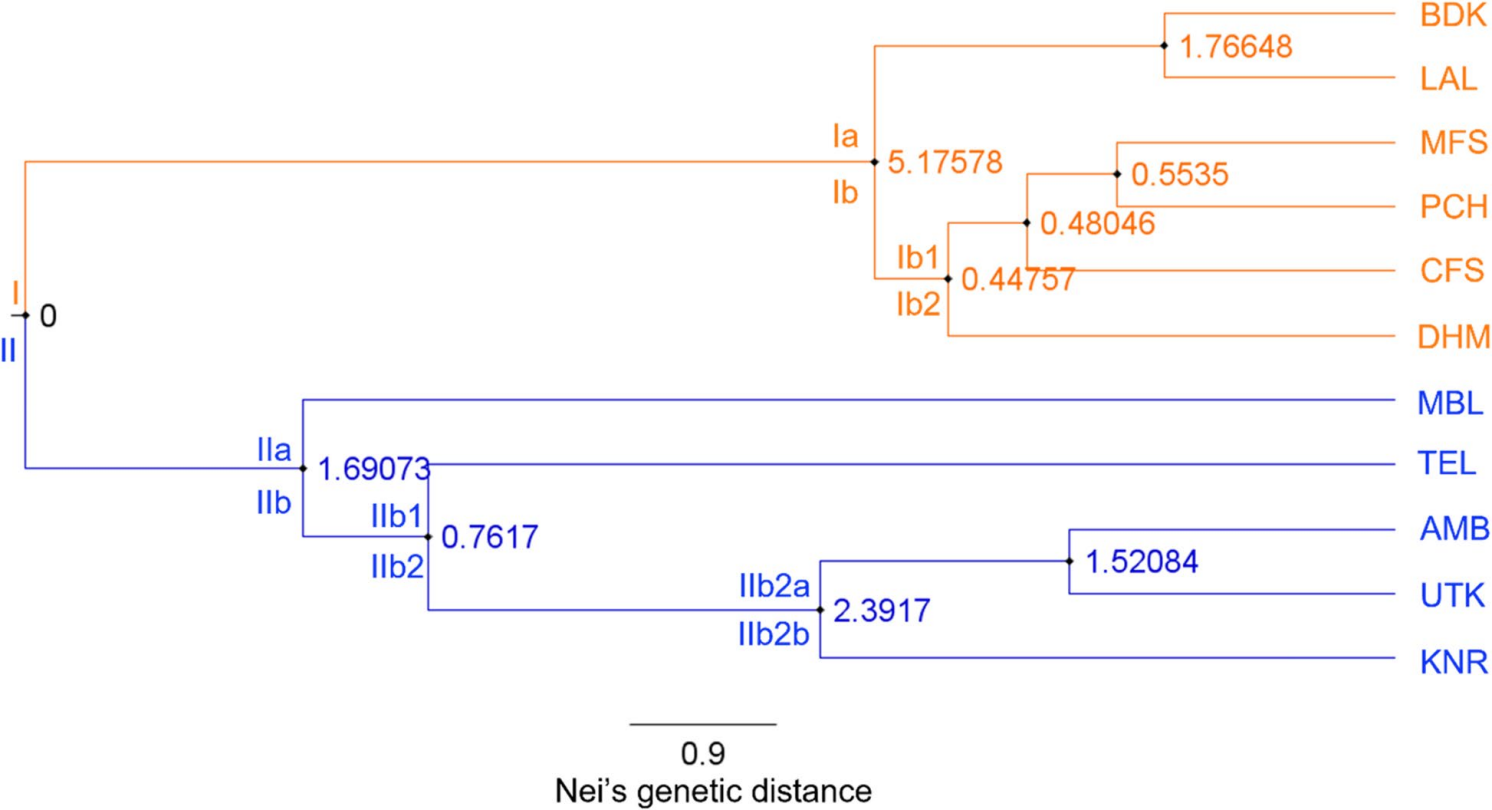
UPGMA dendrogram showing relationships among 11 populations of *G. sylvestre* in India and values at nodes showing branch length. Population codes are as given in the Table 1

**Fig. 3:**
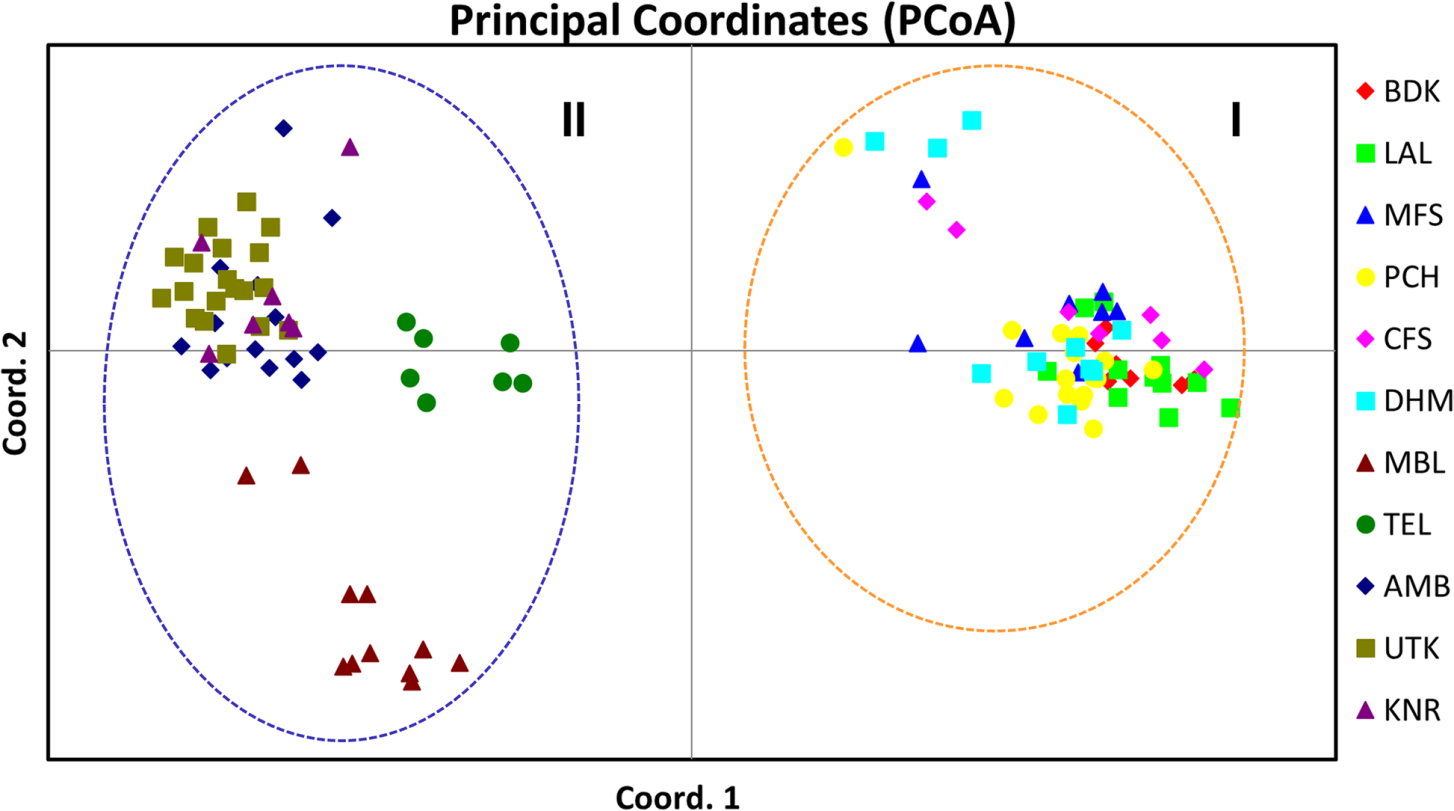
Principal coordinate analysis (PCoA) performed in the program GenAlEx showing clustering of *G. sylvestre* populations in India. The population codes are as given in the Table 1

The UPGMA-based population dendrogram clustered all 11 assumed populations of *G. sylvestre* into two major clusters: cluster I contained all six populations of the North and Central Indian region while cluster II contained all five populations of the South Indian region. In cluster, I, the BDK and LAL populations of the North Indian region are clearly separated out as an independent sub-cluster Ia from the sub-cluster Ib of four Central Indian populations (MFS, PCH, CFS, and DHM). In the sub-cluster Ib, the Chhattisgarh population (DHM) clearly separated out as an independent sub-cluster Ib2 from sub-cluster Ib1 of the rest of the three populations of Madhya Pradesh (MFS, PCH, and CFS). In cluster II, MBL and TEL populations were segregated out as two different independent sub-clusters (IIa and IIb1, respectively) from the rest of the three populations (AMB, UTK, and KNR) comprising sub-cluster IIb2. Furthermore, in this sub-cluster IIb2, the KNR population got segregated as an independent sub-cluster IIb2b from the sub-cluster IIb2a of AMB and UTK populations (Fig. 2). PCoA represented the *G. sylvestre* individuals spatially into three axes corresponding to their genetic distances and grouped all the 118 individuals of 11 *G. sylvestre* populations into two clusters (I and II). Cluster I comprised all 60 individuals of six populations of North and Central India, while cluster II contained rest of the 58 individuals belonging to five populations of South India. The three axes showed 48.62%, 64.59%, and 76.64% cumulative variations, respectively (Fig. 3). Bayesian genetic clustering algorithm of STRUCTURE employed for analyzing population genetic structure revealed two genetic clusters (*K* = 2) of *G. sylvestre* from 11 assumed populations. The cluster I contained all 60 individuals from six natural populations of North and Central India (BDK, LAL, MFS, PCH, CFS, and DHM), which showed an average ancestry membership participation coefficient of 95.5% (BDK = 99.8%, LAL = 99.4%, MFS = 94.4%, PCH = 95.4%, CFS = 93.4%, and DHM = 90.7%) to the inferred cluster I. Cluster II contained all 58 individuals from five natural populations of South India (MBL, TEL, AMB, UTK, and KNR) which showed an average ancestry membership participation of 93.9% (MBL = 95.9%, TEL = 77.1%, AMB = 99.6%, UTK = 99.7%, KNR = 97.2%) to the inferred cluster II. Therefore, the Bayesian assignment of 118 *G. sylvestre* individuals of the 11 assumed geographic populations emerged as the existence of two genetic clusters (Table 6, Fig. S3). The fixation index (*F*_*st*_) which was obtained from STRUCTURE analysis for the two genetic clusters was 0.37 and 0.26, respectively, which indicates that there is adequate genetic differentiation within each genetic cluster.

**Table 6:** Proportion of ancestry membership coefficient of each pre-defined populations in each of the two clusters

Population	Membership to cluster I	Membership to cluster II	Population	Membership to cluster I	Membership to cluster II
BDK	0.998	0.002	MBL	0.041	0.959
LAL	0.994	0.006	TEL	0.229	0.771
MFS	0.944	0.056	AMB	0.004	0.996
PCH	0.954	0.046	UTK	0.003	0.997
CFS	0.934	0.066	KNR	0.028	0.972
DHM	0.907	0.093			
6	0.955 (95.5%)	0.045 (4.5%)	5	0.061 (6.1%)	0.939 (93.9%)

Population codes are as given in Table 1

## Discussion

As the wild populations of *G. sylvestre* are dwindling due to its exploitation for therapeutic uses in traditional and modern medical systems, thorough information on the species genetic diversity and population genetic structure is of utmost priority for its sustainable use, conservation, and management plans. Therefore, the present study was undertaken to analyze the genetic diversity and population structure in 11 representative populations of *G. sylvestre* sampled from the North, Central, and Southern parts of India. The genetic diversity of a species is the product of billions of years of evolution, and it should be studied and protected for a healthy ecosystem and its functions. As the genetic variations do not recover as quickly as population size, it must be preserved appropriately. The varied gene pool of a species allows it to adapt to the changing climatic circumstances and anthropogenic stress so that it can grow and persist in nature, and it is also significant in its environment because it supports the diversity of other related species [61]. Genetic drift, natural selection, mutation, recombination, and gene flow are all important evolutionary mechanisms that influence genetic diversity within a given species and its populations and also influence the genetic structure of a species. A species’ genetic diversity is further governed by its breeding system, seed dispersal technique, life form, geographic range, actual number and sizes of populations, isolation, historical events, ecological and evolutionary history, and anthropogenic activities [61–64].

Genetics has a predominant role in natural population conservation. The increasing importance of genetics in conservation may be attributed to the availability of various kinds of molecular markers, and they also have an impact on our capacity to answer important conservation concerns [61]. Previous comparative studies among DNA marker systems have shown that combining data from all and varied kinds of markers produces the most comprehensive and best findings in a species; therefore, new DNA marker systems are constantly valuable in genetic diversity studies [65, 66]. In plants like *Trichosanthes dioica* and *Mangifera indica*, SPAR (single primer amplification reaction) techniques involving more than one DNA marker system produce more realistic genetic variation [67, 68]. All earlier genetic diversity investigations in *G. sylvestre* were either limited to a narrower geographical area or had fewer individuals or both [33–40]. Using 25 molecular markers (combination of two marker systems: 8 DAMD and 17 ISSR), a comprehensive and cumulative analysis of genetic diversity and population genetic structure of *G. sylvestre* was used in the analyses of genetic diversity in 11 natural populations of *G. sylvestre* which revealed high levels of polymorphism by both the markers independently (DAMD = 82.58% and ISSR = 79.93%) as well as cumulatively (80.89%), indicating that *G. sylvestre* has a broad genetic base. Each marker system was found to be capable of detecting considerable genetic diversity in *G. sylvestre* demonstrating their discriminative efficacy and application in the current investigation. ISSR polymorphism (79.93%) obtained from the present study corroborates with earlier studies carried out in *G. sylvestre* [33, 34, 36]. However, Rathore et al. detected a high level of genetic polymorphisms (ISSR = 97.08%) across 117 individuals of *G. sylvestre* [35]. Present analyses of *G. sylvestre* populations with minisatellite marker system (DAMD marker) showing considerable genetic polymorphisms (82.58%) which is reinforced by the studies conducted in other medicinally important plant species from tropical or sub-tropical climate of India such as *Jatropha curcas* (DAMD = 92.02%) [69], *Musa acuminata* (DAMD = 88.46%) [70], *Curcuma longa* (DAMD = 84.40%) [71], and *Ephedra foliata* (DAMD = 79.70%) [28]. In the present investigation, the DAMD marker system outperformed the ISSR marker system in terms of Rp, EMR, and MI in analyzing the genetic diversity in *G. sylvestre*.

The intra-population genetic diversity analysis for 11 natural populations of *G. sylvestre* revealed that PCH and UTK populations have maximum genetic diversity closely followed by KNR and AMB populations while TEL showed the least genetic diversity. Among the four highly diverse populations, three (UTK, KNR, and AMB) are from the South-Indian region and one (PCH) is from the Central Indian region. The high genetic diversity of these three South-Indian populations (UTK, KNR, and AMB) corroborates with the high diversity found at lower latitudes as compared to higher latitudes. These populations are having lowest three latitudes among all the 11 studied populations. Many previous studies have also come up with the idea that high genetic diversity is found in plants and vertebrates at lower latitudes [72]. Also, their respective climatic condition, i.e., monsoon type with short dry seasons might be supporting their high genetic diversity. And the comparatively big population sizes of UTK and AMB populations are also supporting their high genetic diversity. The high genetic diversity of PCH population from the Central Indian region might be the result of a combination of existing climatic conditions (monsoon type with dry winter) and a big population size. The comparatively least genetic diversity of TEL population might be because of the combination of high temperatures and little precipitation with a small population size. The Indian land has been divided into 9 climate zones viz., “monsoon type with short dry season,” “monsoon type with dry winter,” etc., according to the Köppen’s climate classification system, and these monsoon type climatic conditions accompanied by humidity might be supporting high genetic diversity in plants [73]. As the lianas are mostly found in wet tropical forests, and their abundance and distribution are influenced by rainfall, seasonality, soil fertility, and elevation, therefore, “monsoon-type climatic conditions” are prerequisites for their diversity. Also, in the region-wise genetic diversity estimation, among the three regions, the South Indian region showed maximum genetic diversity closely followed by the Central-Indian region whereas, the North-Indian region showed minimum genetic diversity. In the present investigation, cumulative (8 DAMD + 17 ISSR) marker analysis of 11 *G. sylvestre* populations revealed a considerably high level of genetic diversity at the species level (*H* = 0.26, *I* = 0.40, *PPL* = 80.89%) which is comparable to high genetic diversity indices value of other outcrossing plant species like *Justicia adhatoda* (*H* = 0.25, *I* = 0.39) [74], *Trifolium repens* (*H* = 0.28, *I* = 0.44) [75], and *Asarum mandshuricum* (*H* = 0.28, *I* = 0.42) [76]. In general, outcrossing plant species have significantly higher genetic diversity than selfing species [63, 77]. Nei’s gene diversity value (*H* = 0.26) for *G. sylvestre* was found to be higher than the Nei’s gene diversity calculated for dicotyledons (*H* = 0.17), long-lived perennials (*H* = 0.18), and widespread plants (*H* = 0.18) [64]. The Nei’s genetic diversity value for *G. sylvestre* (*H* = 0.26) is within the range of “0.17–0.33” which has been estimated earlier for outcrossing plants [78]. The value of *H* is also comparable to the previously estimated value of H (by ISSR; *H* = 0.27 and by RAPD; *H* = 0.26) in *G. sylvestre* [35]. Like many other members of the sub-family, Asclepiadoideae, *G. sylvestre* has prominent pollinia and entomophily for cross-pollination, and the most frequent insects for cross-pollination are from the order; Hymenoptera (bees and wasps), Lepidoptera (butterflies and moths), and Diptera (flies) [79]. Besides this, the wind-dispersed seeds (comose seeds) have a significant role in gene flow [33]. Therefore, the considerable prevailing genetic diversity in *G. sylvestre* could be predominantly explained by its life form (long-lived, perennial, liana), breeding system (outbreeding due to conspicuous entomophily), seed (comose seed) wind dispersal mechanism, and geographic range (widely distributed in the entire peninsular Indian region). Generally, long-lived perennials with an outbreeding system, wind dispersal mechanism, and wide geographic spread have a high genetic diversity [64]. Outcrossing species of the Apocynaceae-Asclepiadoideae group have been found to have a high genetic diversity [80]. Although the total genetic diversity for *G. sylvestre* (*H* = 0.26) was found to be remarkably high, the average genetic diversity per population of *G. sylvestre* (*H* = 0.16) was found significantly lower than this total genetic diversity for the species because the average genetic diversity per population gets reduced by differences in genetic diversity by populations. As different populations have to face different climatic conditions, as well as their respective population size also differ from one another so, each population prevails in a different amount of genetic diversity. However, when we compare our estimated genetic diversity of *G. sylvestre* (*H* = 0.26) with other species of its group Apocynaceae; Asclepiadoideae (*Vincetoxicum katoi* and *Vincetoxicum yamanakae*; *H* = 0.31) [81], it seems comparatively less with respect to them. Despite these significant life history traits of *G. sylvestre* which are responsible for its high genetic diversity, the increasing anthropogenic pressure like habitat destruction and fragmentation and global climate change might be reducing its genetic diversity and fitness. Another important feature of *G. sylvestre* which was observed in the wild is that it also produces new progenies by clonal propagation and, in nature, its seed germination rate is also very poor [23]. These two factors also might be responsible for reducing genetic diversity.

Genetic drift, natural selection, recombination, and mutation are the major evolutionary forces responsible for making each population of a species genetically distinct from another population of the same species; therefore, population diversification occurs and over millions of years of time evolution of new species takes place, as these evolutionary forces are helping in maintaining the required genetic differentiation between different populations of a species and also provide the basis for local adaptation to different climatic conditions for each population which is crucial for the evolutionary potential of species. On the other hand, gene flow among populations is the unifying force that binds geographically separated populations into a single evolutionary unit-the species. Without gene flow between populations, populations will become genetically distinct from one another, and if extinction happened to one population, that would result in a significant loss of a particular genetic variation [61]. The value of *G*_*st*_ ranges from zero to one, and the value of *N*_*m*_ ˃ one and ˂ four is considered moderate [26]. The value of Nei’s genetic differentiation among populations (*G*_*st*_ = 0.18) obtained from the present analyses showed low genetic differentiation among 11 different populations of *G. sylvestre* which was created by a moderate rate of migration of genetic materials (*N*_*m*_ = 2.29) among these 11 populations. Being an outcrossing species, the significant gene flow (in the form of pollen and comose seeds) happens to be responsible for lowering the genetic differentiation among populations of species, although it is not proven yet that either entomophily or comose seeds are contributing more towards the gene flow. AMOVA also revealed only 16% of genetic variations present among the populations and most of the genetic variations are present within populations (62%). Results obtained from *G*_*st*_, AMOVA, *H*_*t*_, and *H*_*s*_ showed that of the total genetic variations of the species, most of them are occurring within the populations. In a former study, it was found that the outcrossing plant species with wind dispersal mechanism and long-life spans like *G. sylvestre* has maximum genetic variations within populations while the selfing plant species have maximum genetic variations among populations [77].

The genetic identities and genetic distances between 11 populations of *G. sylvestre* were in congruence with geographical diversity. The IBD model also suggested that the current genetic structure of 11 different populations is in accordance with the geographical distances. Population genetic structure analyses using STRUCTURE resulted into two major genetic clusters (*K* = 2) from the 11 distant populations of *G. sylvestre* suggesting that the moderate amount of gene flow (*N*_*m*_ = 2.29) which has homogenized the nearby populations thus highlighting two major genetic clusters prominently, with an average 5.3% admixing of individuals in which the cluster I showed 4.5% admixing to cluster II and cluster II showed 6.1% admixing to cluster I. Most of the admixing were shown by the peripheral populations like DHM = 9.3% (from North and Central Indian region) and TEL = 22.9% (from South Indian region), respectively. The population genetic structure of *G. sylvestre* with two genetic clusters or two genetic groups (*K* = 2) obtained from the present analyses reveals an intermediate type of species sub-division which is strictly in congruence to the geography and can also be called two geographical groups: the first group of North and Central Indian individuals and the second group of South Indian Individuals. These two genetic clusters show local adaptation to two different geographical areas, respectively, which is good for the evolutionary potential of a species. Within each genetic cluster, there is significant genetic divergence among populations which means all the populations are also locally adapted. The UPGMA and PCoA analyses have supported the result of STRUCTURE as they also came up with two genetic clusters in *G. sylvestre*.

## Conclusions

Genetic polymorphisms from the cumulative data were found to be more appropriate as single type of molecular marker does not provide the best estimate of genome-wide variability in an organism. Comprehensive analyses of genetic diversity and population genetic structure in *G. sylvestre* using cumulative marker data (ISSR + DAMD) with a larger dataset covering a large niche of species were thought to be more informative than previous studies in the species. Considering the medicinal importance of *G. sylvestre*, overexploitation, and depleting populations, different conservation strategies are required for the protection of diverse populations to maintain the species gene pool. Populations like PCH, UTK, KNR, and AMB with maximum genetic diversity should be prioritized for conservation, whereas populations with low genetic diversity, such as MBL and TEL, require adequate management to maximize the diversity. Besides, there is an urgent need to develop promising tissue culture protocols to increase the regeneration process to safeguard existing natural populations and to introduce maximum individuals in impoverished areas to maintain diversity. Results obtained from the present study may be helpful in the identification, collection, and prioritization of genetically varied *G. sylvestre* individuals for its improvement and conservation. The establishment of commercial agro-techniques and standardized harvesting protocols may reduce the pressure on populations of medicinal plants growing in the wild.

## Supplementary Information

**Additional file 1:** **Fig. S1a.** A representative profile of *G. sylvestre* DNA (Gs001 to Gs118) amplified by DAMD primer FVIIex8c. ‘M’ represents a low range DNA ruler (100bp-3kb). Outgroup species DNA is shown at the last (OG). **Fig. S1b.** A representative profile of *G. sylvestre* DNA (Gs001 to Gs118) amplified by ISSR primer UBC-809. ‘M’ represents a low range DNA ruler (100bp-3kb). Outgroup species DNA is shown at the last (OG).

**Additional file 2:** **Fig. S2.** Correlation between geographical and genetic distances of populations of *G. sylvestre* in India, showing geographical distance (in Km) in X axis and Nei’s genetic distance (cumulative marker data) in Y axis.

**Additional file 3:** **Fig. S3.** Bayesian analysis of 118 individuals of *G. sylvestre* in STRUCTURE software showing, **a.** Bar plot representing individuals arranged according to its most likely ancestry **b.** Evanno table highlighting that value of K which describes the genetic groups of species **c.** Graph showing the peak corresponding to that value of K which tells genetic groups of species estimated by Evanno method.

**Additional file 4:** **Table S1.** Mantel Z-statistics carried out for three pairs of data matrices (DAMD, ISSR and cumulative*) in *G. sylvestre*.

**Additional file 5:** **Table S2.** Region-wise diversity statistics of *G. sylvestre* calculated for the three major regions using POPGENE software.

## Data Availability

All data generated or analyzed during this study are included in this published article [and its supplementary information files].

## Abbreviations

DAMD: Directed amplification of minisatellite-region DNA
ISSR: Inter simple sequence repeats
PPL: Percentage of polymorphic loci
PIC: Polymorphic information content
Rp: Resolving power
DI: Diversity index
EMR: Effective multiplex ratio
MI: Marker index
UPGMA: Unweighted pair group method with arithmetic mean
*N*_*a*_: Observed number of alleles
*N*_*e*_: Effective number of alleles
*H*: Nei’s gene diversity
*I*: Shannon information index
*H*_*t*_: Total genetic diversity
*H*_*s*_: Genetic diversity within populations
*G*_*st*_: Nei’s genetic differentiation coefficient among populations
*N*_*m*_: Gene flow among populations
AMOVA: Analysis of molecular variance
PCoA: Principal coordinate analysis
IBD: Isolation by distance
MCMC: Markov chain Monte Carlo
