# Supplementary material for: Estimation of genetic diversity and population genetic structure in Gymnema sylvestre (Retz.) R. Br. ex Schult. populations using DAMD and ISSR markers
Source: J Genet Eng Biotechnol. 2023 Apr 6;21:42. doi: 10.1186/s43141-023-00497-7 (PMC10079795; doi:10.1186/s43141-023-00497-7)
Supplement: Supplementary file 1 — Additional file 1: Fig. S1a. A representative profile of G. sylvestre DNA (Gs001 to Gs118) amplified by DAMD primer FVIIex8c. ‘M’ represents a low range DNA ruler (100bp-3kb). Outgroup species DNA is shown at the last (OG). Fig. S1b. A representative profile of G. sylvestre DNA (Gs001 to Gs118) amplified by ISSR primer UBC-809. ‘M’ represents a low range DNA ruler (100bp-3kb). Outgroup species DNA is shown at the last (OG). [file 43141_2023_497_MOESM1_ESM.docx]

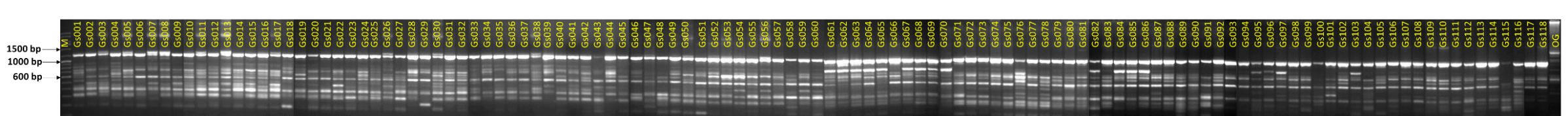


**Fig. S1a** A representative profile of *G. sylvestre* DNA (Gs001 to Gs118) amplified by DAMD primer FVIIex8c. ‘M’ represents a low range DNA ruler (100bp-3kb). Outgroup species DNA is shown at the last (OG)


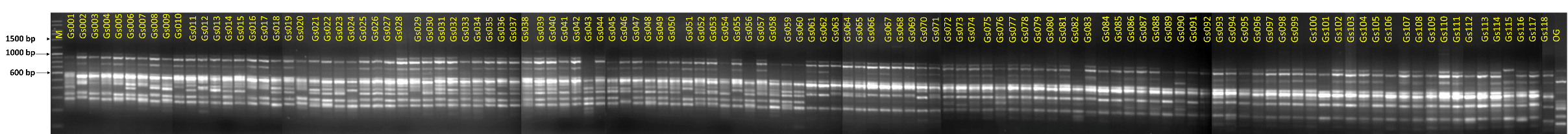


**Fig. S1b** A representative profile of *G. sylvestre* DNA (Gs001 to Gs118) amplified by ISSR primer UBC-809. ‘M’ represents a low range DNA ruler (100bp-3kb). Outgroup species DNA is shown at the last (OG)
