# Supplementary material for: Estimation of genetic diversity and population genetic structure in Gymnema sylvestre (Retz.) R. Br. ex Schult. populations using DAMD and ISSR markers
Source: J Genet Eng Biotechnol. 2023 Apr 6;21:42. doi: 10.1186/s43141-023-00497-7 (PMC10079795; doi:10.1186/s43141-023-00497-7)
Supplement: Supplementary file 2 — Additional file 2: Fig. S2. Correlation between geographical and genetic distances of populations of G. sylvestre in India, showing geographical distance (in Km) in X axis and Nei’s genetic distance (cumulative marker data) in Y axis. [file 43141_2023_497_MOESM2_ESM.docx]

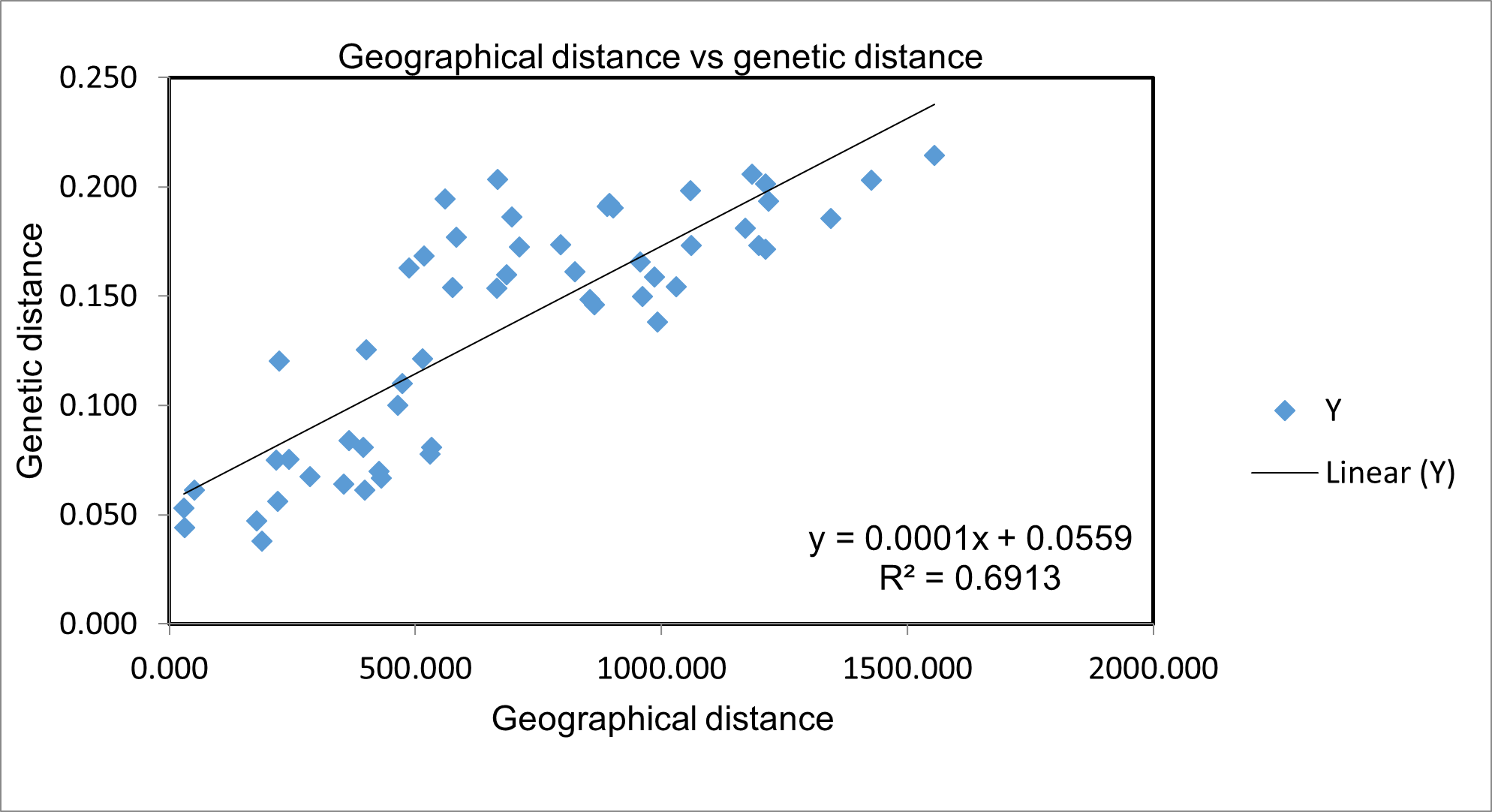


**Fig. S2** Correlation between geographical and genetic distances of populations of *G. sylvestre* in India, showing geographical distance (in Km) in X axis and Nei’s genetic distance (cumulative marker data) in Y axis
