# Supplementary material for: Estimation of genetic diversity and population genetic structure in Gymnema sylvestre (Retz.) R. Br. ex Schult. populations using DAMD and ISSR markers
Source: J Genet Eng Biotechnol. 2023 Apr 6;21:42. doi: 10.1186/s43141-023-00497-7 (PMC10079795; doi:10.1186/s43141-023-00497-7)
Supplement: Supplementary file 3 — Additional file 3: Fig. S3. Bayesian analysis of 118 individuals of G. sylvestre in STRUCTURE software showing, a. Bar plot representing individuals arranged according to its most likely ancestry b. Evanno table highlighting that value of K which describes the genetic groups of species c. Graph showing the peak corresponding to that value of K which tells genetic groups of species estimated by Evanno method. [file 43141_2023_497_MOESM3_ESM.docx]

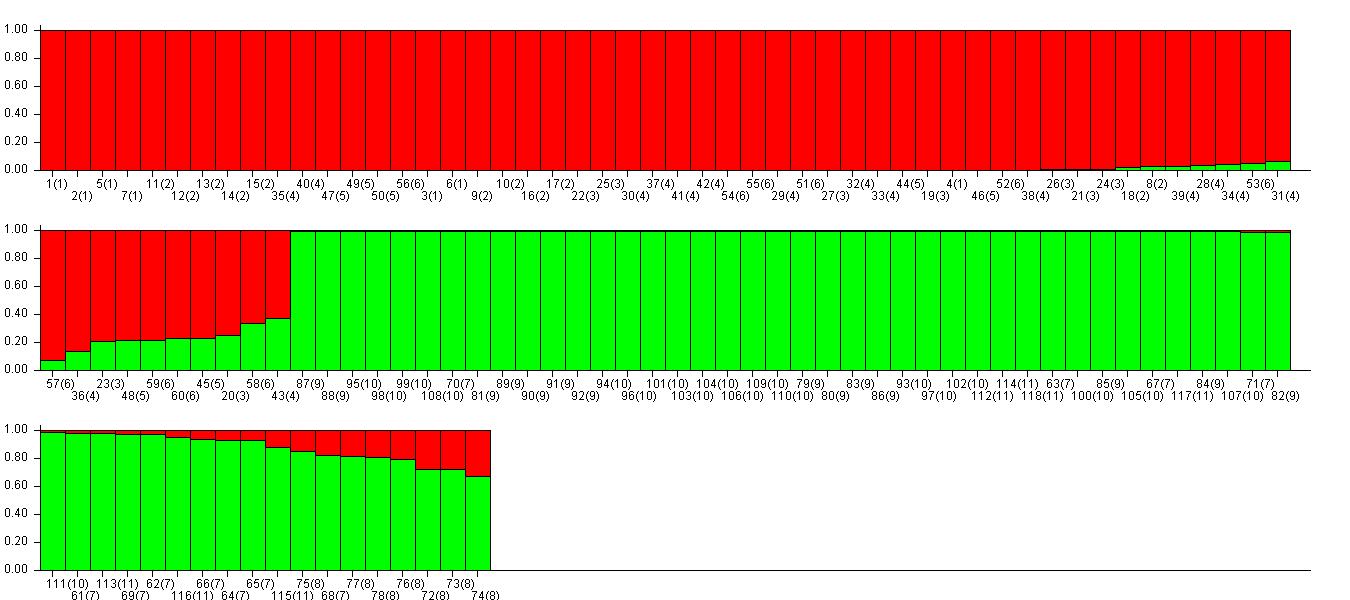


**CLUSTER I**

**CLUSTER II**

**a.**

| K | Reps | Mean LnP(K) | Stdev LnP(K) | Ln'(K) | \|Ln''(K)\| | Delta K |
| --- | --- | --- | --- | --- | --- | --- |
| 1 | 20 | -37630.005000 | 0.890579 | — | — | — |
| 2 | 20 | -31854.235000 | 8.502772 | 5775.770000 | 4257.505000 | 500.719648 |
| 3 | 20 | -30335.970000 | 190.460301 | 1518.265000 | 335.010000 | 1.758949 |
| 4 | 20 | -29152.715000 | 431.454733 | 1183.255000 | 32.765000 | 0.075941 |
| 5 | 20 | -28002.225000 | 341.516705 | 1150.490000 | 1991.020000 | 5.829934 |
| 6 | 20 | -28842.755000 | 3713.098518 | -840.530000 | 3104.140000 | 0.835997 |
| 7 | 20 | -26579.145000 | 171.552004 | 2263.610000 | 1830.290000 | 10.669010 |
| 8 | 20 | -26145.825000 | 390.125089 | 433.320000 | 3686.705000 | 9.450059 |
| 9 | 20 | -29399.210000 | 9014.790263 | -3253.385000 | 4012.245000 | 0.445074 |
| 10 | 20 | -28640.350000 | 6474.118122 | 758.860000 | 8448.605000 | 1.304982 |
| 11 | 20 | -36330.095000 | 42196.462783 | -7689.745000 | 18981.335000 | 0.449832 |
| 12 | 20 | -25038.505000 | 1374.395045 | 11291.590000 | — | — |

**b.**


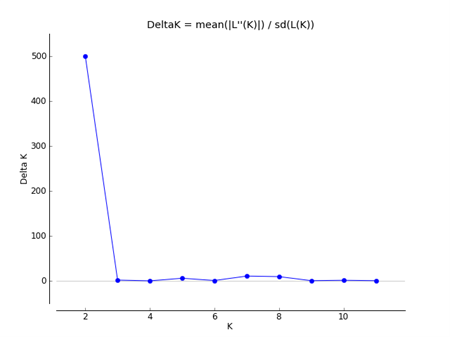


**c.**

**Fig. S3** Bayesian analysis of 118 individuals of *G. sylvestre* in STRUCTURE software showing, **a.** Bar plot representing individuals arranged according to its most likely ancestry **b.** Evanno table highlighting that value of K which describes the genetic groups of species **c.** Graph showing the peak corresponding to that value of K which tells genetic groups of species estimated by Evanno method
