# Supplementary material for: Estimation of genetic diversity and population genetic structure in Gymnema sylvestre (Retz.) R. Br. ex Schult. populations using DAMD and ISSR markers
Source: J Genet Eng Biotechnol. 2023 Apr 6;21:42. doi: 10.1186/s43141-023-00497-7 (PMC10079795; doi:10.1186/s43141-023-00497-7)
Supplement: Supplementary file 4 — Additional file 4: Table S1. Mantel Z-statistics carried out for three pairs of data matrices (DAMD, ISSR and cumulative*) in G. sylvestre. [file 43141_2023_497_MOESM4_ESM.docx]

**Table S1** Mantel Z-statistics carried out for three pairs of data matrices (DAMD, ISSR and cumulative*) in *G. sylvestre*

| Marker Pairs | Matrix correlation coefficient (*r*) | Mantel t-test (*t*) | Probability (*p*) |
| --- | --- | --- | --- |
| DAMD v/s cumulative* | 1.00 | 48.43 | 1.00 |
| ISSR v/s cumulative* | 1.00 | 48.43 | 1.00 |
| DAMD v/s ISSR | 1.00 | 30.36 | 1.00 |

*Combined DAMD and ISSR data matrices
