# Supplementary material for: Estimation of genetic diversity and population genetic structure in Gymnema sylvestre (Retz.) R. Br. ex Schult. populations using DAMD and ISSR markers
Source: J Genet Eng Biotechnol. 2023 Apr 6;21:42. doi: 10.1186/s43141-023-00497-7 (PMC10079795; doi:10.1186/s43141-023-00497-7)
Supplement: Supplementary file 5 — Additional file 5: Table S2. Region-wise diversity statistics of G. sylvestre calculated for the three major regions using POPGENE software. [file 43141_2023_497_MOESM5_ESM.docx]

**Table S2** Region-wise diversity statistics of *G. sylvestre* calculated for the three major regions using POPGENE software

| Region (SS) | Mean *N_a_* (SD) | Mean *N_e_* (SD) | Mean *H* (SD) | Mean *I* (SD) | NPL | *PPL* | Mean *H_t_* (SD) | Mean *H_s_* (SD) | *G_st_* | *N_m_* |
| --- | --- | --- | --- | --- | --- | --- | --- | --- | --- | --- |
| North India (18) | 1.47 (0.50) | 1.26 (0.35) | 0.15 (0.19) | 0.23 (0.28) | 201 | 46.85 | 0.15 (0.04) | 0.14 (0.03) | 0.10 | 4.27 |
| Central India (42) | 1.69 (0.46) | 1.36 (0.36) | 0.22 (0.19) | 0.33 (0.27) | 296 | 69.00 | 0.21 (0.04) | 0.18 (0.03) | 0.17 | 2.46 |
| South India (58) | 1.73 (0.45) | 1.39 (0.35) | 0.24 (0.19) | 0.36 (0.27) | 312 | 72.73 | 0.24 (0.04) | 0.16 (0.02) | 0.31 | 1.11 |

*SS* sample size, *N_a_* observed number of alleles, *N_e_* effective number of alleles, *H* Nei’s gene diversity, *I* Shannon’s information index, *NPL* number of polymorphic loci, *PPL* percentage of polymorphic loci*, H_t_* total genetic diversity, *H_s_* genetic diversity within populations, *G_st_* Nei’s genetic differentiation coefficient among populations, *N_m_* gene flow among populations, *SD* standard deviation of mean values
